# Molecular Mechanisms of Human Papillomavirus Induced Skin Carcinogenesis

**DOI:** 10.3390/v9070187

**Published:** 2017-07-14

**Authors:** Martin Hufbauer, Baki Akgül

**Affiliations:** Institute of Virology, University of Cologne, Fürst-Pückler-Str. 56, 50935 Cologne, Germany; martin.hufbauer@uk-koeln.de

**Keywords:** *betapapillomavirus*, extracellular matrix, invasion, cancer initiating cells, wound healing, squamous cell carcinoma

## Abstract

Infection of the cutaneous skin with human papillomaviruses (HPV) of genus *betapapillomavirus* (βHPV) is associated with the development of premalignant actinic keratoses and squamous cell carcinoma. Due to the higher viral loads of βHPVs in actinic keratoses than in cancerous lesions, it is currently discussed that these viruses play a carcinogenic role in cancer initiation. In vitro assays performed to characterize the cell transforming activities of high-risk HPV types of genus *alphapapillomavirus* have markedly contributed to the present knowledge on their oncogenic functions. However, these assays failed to detect oncogenic functions of βHPV early proteins. They were not suitable for investigations aiming to study the interactive role of βHPV positive epidermis with mesenchymal cells and the extracellular matrix. This review focuses on βHPV gene functions with special focus on oncogenic mechanisms that may be relevant for skin cancer development.

## 1. Introduction

Keratinocyte derived squamous cell carcinoma (SCC) is the most common metastatic skin cancer, and its incidence is increasing worldwide [1]. Exposure to ultraviolet (UV) radiation is accepted to be the main risk factor for skin carcinogenesis. Most skin SCC arise in association with a distinct precancerous lesion, the actinic keratosis. Epidemiological data indicate that human papillomaviruses (HPV) of genus *betapapillomavirus* (βHPV) may have a co-factorial role in this process [2,3,4,5]. The oncogenic potential of βHPV in skin carcinogenesis was originally identified in patients suffering from the rare inherited disease Epidermodysplasia verruciformis (EV), who have an increased susceptibility to βHPV infections. These viruses can also be found in skin cancers of non-EV patients. Also, immunosuppressed organ-transplant-recipients (OTR) have a higher susceptibility to βHPV infection in the skin as well as an increased risk of developing SCC compared with healthy individuals [6,7,8]. However, the association of βHPV infection and skin SCC development in the normal population remains controversial. Here, higher βHPV prevalence rates and viral loads can only be found in precancerous actinic keratoses but are missing in SCC [9]. These observations, however, are compatible with a carcinogenic role of these viruses in skin cancer initiation, probably through a hit-and-run mechanism where the virus acts as a co-factor along with UV to promote driver mutations in stem cells.

In contrast to high-risk *alphapapillomaviruses* (αHPV), that are causative factors for the development of cervical, anal, and oropharyngeal cancers [10], the molecular mechanisms that underlie the role of βHPV types in SCC development are less well understood. The specific tropism of cutaneous and mucosal HPV strains suggests that the life cycle is tied to particular epithelial cell types, which implies that cancer models for cutaneous HPVs cannot be unrestrictedly adopted from those for mucosal types. In vitro studies performed with conventional submerged keratinocyte cultures have markedly contributed to the present knowledge on the keratinocyte transforming activities by αHPV. However, these in vitro assays were not suitable for investigations aiming to study the interactive role of βHPV positive keratinocytes with mesenchymal cells and the extracellular matrix (ECM), as the spatial tissue organization in monolayer cultures is missing. This review highlights the recent progress in the field and outlines some unresolved questions related to oncogenic functions of βHPV during skin cancer initiation. 

## 2. Invasion is Regulated Both at the Level of the Tumor Cell and the Extracellular Matrix

Alterations in the microenvironment of keratinocytes are required for initiation and progression over the course of cancer development. Difficulties in generating differentiating epithelia in vitro have hampered molecular studies involving cutaneous types and the extracellular matrix. Three dimensional organotypic skin cultures are useful systems for the in vitro analysis of skin biology because they mimic keratinocyte differentiation far better than monolayer cultures [11,12]. These experimental setups employ human cutaneous keratinocytes and a mesenchymal matrix that is repopulated with dermal fibroblasts. Cellular functions requiring epithelial differentiation, cell and extracellular matrix interactions, or keratinocyte-fibroblast paracrine communications can be observed and analyzed to a much higher degree. Boxman et al. [13] were the first to analyze the potential cell transforming activities of cutaneous HPV types in such an organotypic skin culture model. To study the effect of cutaneous HPV gene expression on keratinocytes, organotypic cultures based on a collagen type I matrix were repopulated with 3T3 mouse fibroblasts and primary keratinocytes expressing the *E6/E7* genes of βHPV types HPV5, 12, 15, 17, 20, and 38 as well as the high-risk αHPV type HPV16. These cultures showed varying degrees of dysregulated keratinocyte differentiation but lacked the features of cancer progression including the key step of basement membrane invasion for any of the HPV types analyzed. However, collagen-based organotypic cultures seem to mimic an in vivo environment in which invasion-specific βHPV oncoprotein functions cannot be carried out. In light of this observation, it needs to be noted that such exclusively collagen-comprised organotypic cultures lack the epidermal basement membrane and other ECM proteins. Over the last several years, it has become increasingly evident that tumor development requires cross-talk between different cell types within the tumor and its surrounding stroma. The latter provides a connective tissue, which is believed to possess a defined composition of structural and cellular components [14]. The knowledge of the most important constituents of the extracellular matrix, their metabolism and degradation provides insight into the pathophysiology of malignant growth [15]. With the published data of Boxman and colleagues in mind [13], we set out to investigate the effects of E6 and E7 of the βHPV type 8 (HPV8) in a more physiological skin-equivalent three dimensional (3D) model which sets itself apart from other skin culture models by using a de-epidermalized human dermis repopulated with either *E6* or *E7* positive cutaneous keratinocytes. Under these conditions, HPV8-E6 positive regenerated skin showed less epithelial layers compared to matched controls, which indicated at that time that E6 inhibits the normal differentiation program in keratinocytes [16,17]. In particular HPV8-E7 positive keratinocytes displayed significantly altered proliferation and differentiation. These skin cultures displayed enhanced terminal cell differentiation and a hyperproliferative phenotype, as evidenced by increased cornification and the presence of dividing cells in suprabasal layers. In addition and most strikingly, the cells lost their normal polarity and gained the ability to invade the dermal matrix. Migration of keratinocytes downward into the dermis was facilitated by degradation of components of the basement membrane and the extracellular matrix through the induction of the expression of matrix metalloproteinases [18,19]. As a result, basement membrane integrity was compromised in a time-dependent manner as evidenced by the degradation of collagen VII, collagen IV, and laminin-V. These observations revealed the hitherto unknown activity of βHPV—the ability to promote cell invasion. In addition, the accumulated data suggested that understanding of the composition of the tumor stroma and the interaction of βHPV positive keratinocytes with the ECM are important in discerning mechanisms regulating cell invasion. However, the molecular basis regulating the invasion of βHPV positive keratinocytes was still not known.

In the context of carcinoma pathogenesis, the conversion of normal keratinocytes to cancer cells induces an epithelial-mesenchymal-transition (EMT), which is associated with changes in intercellular adhesion molecules [20]. At the molecular level, this involves a reorganization of cell-cell adhesion complexes and modifications in cell-matrix interactions. Related to cell-cell connections, HPV8-E7 possesses the capacity to deregulate cell-cell junctions through the upregulation of β-catenin, zona occuldens protein 1 (ZO-1) [21], and the AKT serine/threonine kinase 2 (AKT2) [17]. Studies based on cell-matrix interactions revealed that the ECM proteins collagen IV, laminin V, and fibronectin all trigger HPV8-E7-mediated invasion. However, invasion associated with an EMT phenotype was only observed on fibronectin matrices. This indicates that ECM proteins can exert different modes of invasion. Most interestingly, fibronectin, but not collagen variants nor laminin-V were found to be deposited in peritumoral areas in HPV8 positive skin SCC that could have been produced and secreted by infected keratinocytes and by keratinocyte-stimulated fibroblasts. Again, only fibronectin led to a shift in the cell surface expression profiles of integrin, which are glycoprotein receptors mediating cell-matrix adhesion [15,22]. Invasive keratinocytes showed enhanced cell surface localization of integrin α3β1. Silencing of the α3 chain or using the 8E7 mutant L23A which is incapable of inducing invasion, prevented keratinocyte invasion, thus providing evidence for a role of the α3β1 integrin and fibronectin interplay in the invasion of HPV8 expressing keratinocytes [23]. Sonnenberg’s group identified that epidermal-specific deletion of α3β1 integrin leads to a reduction of skin tumorigenesis in mice. However, tumors that did form progressed more rapidly to invasive carcinoma, implying a requirement for elevated α3β1 cell surface presence during tumor initiation and early growth [24]. These findings and our observations provide some evidence that α3β1 integrin and fibronectin may represent mediators of invasion during the initiation of the invasion cascade by βHPV.

Unfortunately, the answer how fibronectin and α3β1 integrin regulate keratinocyte invasion is not clear as yet because α3β1 is mainly considered to be a laminin-V receptor. It also needs to be noted that increased invasion often results from reduced adhesion to the ECM [25,26]. Furthermore, previous investigative efforts to ascertain a potential fibronectin binding by α3β1 have been contradictory. However, studies showing minimal binding of α3β1 to fibronectin were performed on cells cultured on plastic, whereas several other studies confirming this interaction utilized ECM interaction assays [27]. Yet many of these studies employed cell-based attachment assays, in which the binding affinity of α3β1 to ECM ligands was influenced in the presence of additional integrins [28,29]. Studies on the cross-talk between α3β1 with other fibronectin binding integrins may shed light on the underlying mechanism leading to keratinocyte invasion. 

## 3. Mouse Models

Further in vivo evidence for the cell transforming ability of HPV8 early proteins was provided with the generation of HPV8 transgenic mice. Nearly all transgenics containing the complete early genome region of HPV8 under the control of the human keratin-14 promoter (K14-HPV8-CER) developed papillomas, partially with moderate or severe dysplasia. In 6% of the animals, SCC developed without previous exposure to physical or chemical carcinogens [30,31]. A mechanistic link between HPV8 protein expression and UV exposure in the development of skin tumors could be established by induction of skin papillomas in UVA/B treated animals in about three weeks after treatment [32,33]. Proteolytic activity identified in the peritumoral stroma and epidermal sheets hinted at a cross-talk between HPV8 positive keratinocytes and stromal cells [31]. Cross-breeding K14-HPV8-CER animals with *Stat3* heterozygous animals or mice with epidermal knock-out of *Rac1* inhibited papilloma formation. This pointed to an important role of Stat3- and Rac1-dependent pathways in HPV8 induced tumorigenesis [34,35]. By expressing individual HPV8 proteins under the control of the keratin14 promoter, the tumorigenic potential of the individual viral proteins could be analyzed in greater detail. HPV8-E2 mice presented mild to severe skin dysplasia mostly in their second year of life [36]. The kinetics of tumor development in K14-HPV8-E6 mice were comparable to K14-HPV8-CER mice [37]. This and the observation that E6 expression is necessary and sufficient for induction of papilloma formation [32] pointed to E6 as the major oncogene of HPV8 in the murine epidermis. While K14-HPV8-E6wt mice developed papillomas within three weeks post UV irradiation, skin tumor formation was significantly inhibited in mice, in which the E6 mutant K136N was expressed (K14-HPV8-E6K136N) which, in contrast to E6wt, is unable to impair DNA damage repair. These results provided further evidence for an interplay of UV-light and βHPV gene expression [38]. K14-HPV8-E7 animals showed no papilloma formation despite very low transgene expression. However, they exhibited carcinoma in situ formation early after chronic UVA/B treatment [23]. In conclusion, studies performed on HPV8 as well as HPV38 transgenic mice [39,40] uncovered several oncogenic functions of βHPV early proteins and represent suitable models to mimic high viral early gene expression as seen in EV or in OTR patients. However, to establish a possible hit-and-run mechanism of tumorigenesis it would be a required prerequisite to establish suitable animal models where the kinetics of βHPV gene expression and disease onset can be studied with translational applicability to the normal human population. Animals with βHPV genes under the control of inducible promoters may represent appropriate models for mechanistic evaluations on HPV oncogene functions in early phases of skin cancer development. In addition, the multimammate rat Mastomys coucha, whose skin is naturally infected—similar to βHPV in humans—with the Mastomys natalensis papillomavirus (MnPV) [41,42] may provide an animal model in which tumorigenic processes can be studied with a background of the complete viral life cycle. 

## 4. Do βHPV Affect Self-Renewal of Infected Keratinocytes?

An apparent prerequisite for cancer initiation is the property to generate cancer stem cells [43]. Epidermal stem cells are located either in the basal epidermis or in the hair follicles. Like all stem cells, they are able to undergo asymmetric cell division, giving rise to one true stem cell which ensures that their population is not depleted, and a second, transiently amplifying cell that maintains and further amplifies along its differentiation axis before undergoing terminal differentiation [44]. Since βHPV were consistently detected on plucked hairs, it is postulated that the natural reservoir for βHPV latent infection resides within the hair follicular stem cells [45,46,47]. Considering that in skin SCC only a minority of cells harbor βHPV and viral functions are not required for cancer maintenance, a “hit-and-run” mechanism would be an explanation for the contribution of βHPV to skin cancer development. This implies that the expansion of the tumor is driven by virally-infected cancer stem cells, in which UV-induced driver mutations accumulated are caused by the interference of the viral E6 protein with the DNA repair mechanisms [38,48,49]. The marked decrease in the number of apoptotic cells in βHPV positive SCC compared to HPV negative cancers, and the continued expression of proliferation markers [50,51], indicates that the balance between apoptosis and proliferation is modified in βHPV-containing lesions. This may be the result of a cellular cross-talk between the βHPV positive stem cells and normal neighboring keratinocytes. The identification of soluble mediators or still unknown mechanisms may aid in our efforts to understand how βHPV positive stem cells communicate with and modulate their cellular environment.

**Figure 1:**
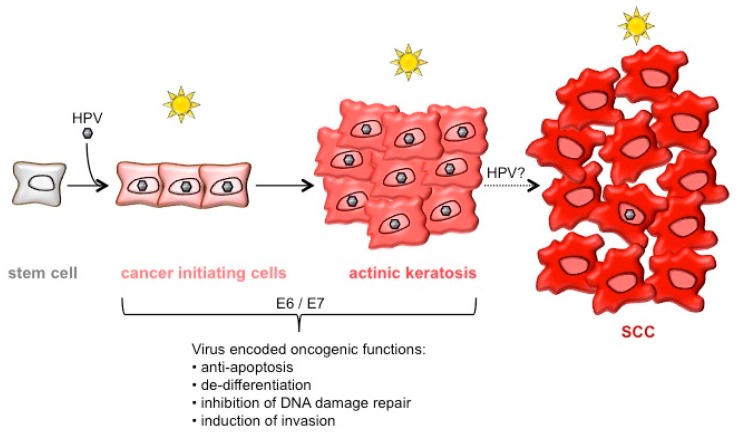
Model of keratinocyte transformation stages that may represent initial phases of *betapapillomavirus* (βHPV)-induced squamous cell carcinoma (SCC) development. The accepted risk factor for SCC development is ultra-violet (UV)-irradiation. Infection with βHPV may play a carcinogenic role in the early phases of SCC development. The human papillomavirus (HPV)-mediated expansion of the stem cell pool may allow the generation of cancer-initiating stem cells, which can give rise to actinic keratoses. Yet unknown is the exact role of HPV in the progression of actinic keratoses to SCC, in which only few cells harbor viral genomes.

In a very recent study, Marisa Gariglio and her team addressed the role of different hair follicle stem cells in HPV8-induced skin cancer development utilizing K14-HPV8-CER mice [52]. They identified the leucine rich repeats and immunoglobulin like domains 1 (Lrig1) positive stem cell population, residing in the hair follicle junctional zone [53], to be expanded in HPV8 transgenic animals. Proliferation in these cells is induced by the overexpression of the p63 protein lacking the N-terminal domain (ΔNp63) that is accepted as an epidermal stemness marker [52]. At least for HPV8-E6, it is known to trigger ΔNp63 levels [54,55]. It still needs to be determined whether other HPV8 early proteins also target ΔNp63 and which viral protein is responsible for the expansion of the Lrig1 positive stem cell population. Our group has previously shown that HPV8 early proteins can increase the number of keratinocytes with stem cell-like properties in monolayer culture. In these assays, HPV8-E7 in particular increased the clonogenicity of transduced keratinocytes and led to the formation of tumor spheres, which are generally regarded as cancer stem cells with self-renewal and tumorigenic capacities. Stem cell-like characteristics were associated with an increase in the epidermal stemness markers cluster of differentiation (CD)44 and epithelial cell adhesion molecule (EpCAM) [56]. Interestingly, the α3 integrin chain, crucial for the regulation of cell invasion, is known to be co-expressed with CD44 and EpCAM on tumor cell lines [57] and was indeed expressed in higher levels on *E7* positive cells with a CD44high/EpCAMhigh immunophenotype. Accumulating evidence indicates that EMT processes contribute to the progression of several carcinoma types (reviewed in reference [20]) and result in the generation of epithelial cells with stem-like properties [58]. It remains to be seen whether the growth of βHPV oncoprotein expressing keratinocytes on a fibronectin matrix may trigger cells to enter faster into this state. In line with the established fact that βHPV types (including HPV8) inhibit keratinocyte differentiation, apoptosis and DNA damage repair processes [38,55,59,60], these observations indicate that βHPV may increase the pool of cells in which DNA damage can persist and lead to the generation of stem cells with malignant properties. Additional experimental evidence is required to address and clarify more precisely the events occurring in βHPV positive stem cells. The identification and molecular characterization of these βHPV positive keratinocytes may aid in understanding the hit-and-run mechanism of βHPV-initiated skin cancer development (Figure 1). A precise understanding of the molecular program involved in regulating self-renewal and long-term survival in these cells may lead to potential drug targets and is therefore also of clinical relevance.

## 5. Do Wound-Healing Processes Play a Role in βHPV-Mediated Cancer Initiation?

**Figure 2:**
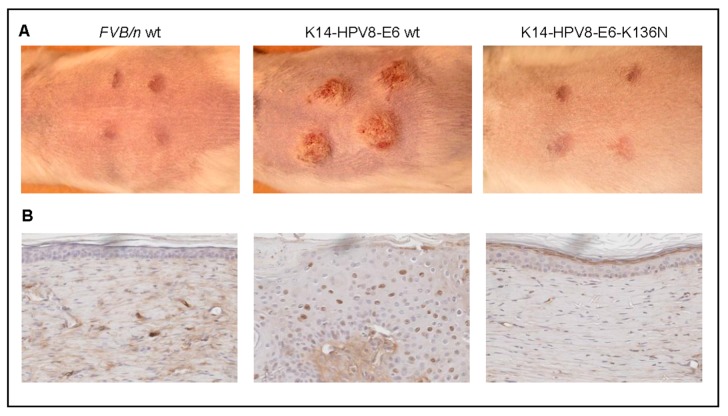
Persistence of DNA damage in full-thickness wounded K14-HPV8-E6 wt mice. Five weeks old *FVB/n* wt, K14-HPV8-E6 wt and K14-HPV8-E6K136N mice (*n* = 8 for each mouse line) were anaesthetized and wounded dorsal-caudal. Four punch biopsies were taken from the skin of the back creating four circular 4-mm wounds according to previously published procedures [37]. (**A**) Representative macroscopical images of animals taken 24 days after full-thickness wounding; (**B**) Paraffin embedded sections of from treated skin areas were stained for γH2AX (clone EP854(2)Y, Millipore (Darmstadt, Germany), 1:750 dilution used overnight). Sections were developed using the Vectastain Elite ABC kit (Linaris, Dossenheim, Germany; brown staining) and counterstained with haematoxylin (violet staining) (magnification: 400×). These experiments were approved by the governmental animal care office North-Rhine-Westphalia (approval no. 8.87-50.10.35.08.163).

Another open question is the issue of whether signals that result in basal keratinocyte activation, spreading, and migration during cutaneous wound re-epithelialization may also contribute to βHPV-mediated cancer initiation. It is well established that tissue repair relies on stem cells and that chronic wounds predispose for tumor formation [61]. Such associations, together with similarities in the histology of wounds and tumors, led Dvorak to the often-cited conclusion that “tumors are wounds that do not heal” [62]. Several lines of evidence indicate that wound-healing processes may contribute to βHPV-induced cancer initiation as well:

(1) K14-HPV8-CER mice spontaneously developed papillomas and skin SCC without any treatment with physical or chemical carcinogens. Interestingly, these spontaneous skin lesions arose mostly in places where the animals had also scratched themselves. These results led to the hypothesis that activated keratinocytes that drive re-epithelialization fail to switch off the activated status upon finalized wound-healing and do not return to their normal differentiation pathway. 

(2) While full-thickness wounding induced papilloma growth at the wound sites in 100% of K14-HPV8-E6wt animals, in marked contrast, none of the *FVB/n* control nor of K14-HPV8-E6K136N mice developed lesions following wounding (Figure 2A). It has been demonstrated that wound-healing-induced oxidative stress and the resulting reactive oxygen species (ROS) can affect the transcription of DNA damage repair enzymes. Yet, it may also directly inactivate DNA repair by oxidation. ROS can induce a number of modifications to DNA including single strand breaks, double strand breaks (DSBs), strand cross-links, along with protein-DNA cross-links and therefore increase the rate of mutations which may contribute to tumorigenesis [63]. We found that phosphorylation of the Ser-139 residue of the histone variant H2AX, forming γH2AX, a surrogate marker for DSB, were present in the skin tumors of K14-HPV8-E6wt mice taken 24 days after full-thickness wounding but not in healed skin of *FVB/n* and K14-HPV8-E6K136N animals (Figure 2B). These results support the hypothesis that wound-healing processes together with βHPV oncogene expression may contribute to the persistence of DSB and consequently to skin tumorigenesis.

(3) Tumor progression in HPV8 transgenic mice was paralleled by a strong inflammatory response within the tumor stroma, dominated by macrophages promoting tumor initiation and progression. In addition, wounding-associated tumor initiation was prevented after epidermal-specific depletion of vascular endothelial growth factor (VEGF) in HPV8 transgenic mice [64].

(4) The HPV8 target proteins fibronectin and α3β1 integrin are critical factors regulating epithelial repair during wound-healing [65,66,67].

The understanding of the similarities among wounding and UV-induced skin tumors and how βHPV-induced oxidative stress interferes with normal DNA damage repair may yield further insights into the mechanisms behind the impaired DNA damage response that may drive cancer initiation in infected cells. 

## 6. Conclusions

While much of the underlying general mechanisms of βHPV-initiated keratinocyte transformation are unknown, recent discoveries have aided in directing future work towards the molecular basis of βHPV oncogenesis. We still need to understand the molecular alterations of how βHPV-mediated clonal expansion of cells with enriched UV signature mutations can result in the formation of pre-cancerous lesions and drive the progression to SCC in the absence of the virus. Since several βHPV types are potentially involved in the development of skin SCC, future research may hopefully identify more general oncogenic mechanisms of βHPV—in addition to the known genus-specific targeting of Mastermind-like protein-1 (MAML1) by E6 [68,69,70] and pRb, UBR4, KCMF1, and PTPN14 by E7 [69,71,72]—that underline the proposed hit-and-run mechanism of tumorigenesis.
